# Impacts of Haze on Housing Prices: An Empirical Analysis Based on Data from Chengdu (China)

**DOI:** 10.3390/ijerph15061161

**Published:** 2018-06-02

**Authors:** Runqiu Liu, Chao Yu, Canmian Liu, Jian Jiang, Jing Xu

**Affiliations:** 1 Institute of Land Economics and Land Management, Sichuan University, Chengdu 610065, China; liurq@scu.edu.cn; 2 School of Public Administration, Sichuan University, Chengdu 610065, China; 2015225010159@stu.scu.edu.cn (C.Y.); 2017225010138@stu.scu.edu.cn (J.J.); 3 Business School, Sichuan University, Chengdu 610065, China; 2016225025022@stu.scu.edu.cn; 4 The Economy and Enterprise Development Institute, Sichuan University, Chengdu 610065, China

**Keywords:** haze, air quality, housing prices, spatial error model, spatial lag model, hedonic price model

## Abstract

Based on cross-section data of 20 districts in Chengdu, this article reviews the relationships between haze and housing prices with the combined application of Spatial Error Model (SEM) and Spatial Lag Model (SLM). The results illustrate that haze significantly have negative impacts on both the selling and rental prices of houses. Controlling other variables, if the air quality index rises by 0.1, the housing selling prices and rental prices will drop by 3.97% and 4.01%, respectively. Interestingly, housing rental prices have a more significant response to the air quality than housing sale prices. Residents are willing to pay a premium for better air quality and the influence of air quality is partially reflected in housing prices, which indicates that better air quality has been becoming a scarce resource with the improvement of people’s living standard. Furthermore, the impacts of haze on housing prices are also expected to lead to a “crowding out effect” in different regions. This would be detrimental for human capital accumulation and will accelerate the regional divergence in the internal economy and population structure, thus forming a region “fence” within cities.

## 1. Introduction

Air pollution has become a major issue in China, and haze has become a serious pollution issue as a result. With the improvement in environmental awareness and healthy life quality among the general population, air quality problems are expected to gradually affect the flow of social talents and economic elements in a society. Certainly, the aggravation of haze pollution will further strengthen its influence on the flow of social resources. In 2016, the article “*Southwest China Position Paper 2015/2016*”, issued by the European Union Chamber of Commerce in China, pointed out that “Many experienced foreign investors with technical expertise and experience will consider moving to less polluted areas if Chengdu’s air pollution can’t be solved properly” [1]. This reflects the impacts of haze on the residential areas that people choose to live in; air quality guides residents’ choices of housing. People living in the city have to consider how to find relatively healthy residences in a city severely impacted by haze and, as a result, people tend to choose their living areas according to their earnings and preferences for air quality. This in turn means that air quality is capitalized to housing prices. This issue has raised widespread social discussions and concerns.

## 2. Literature Review

There have been many studies on the relationship between air quality and housing prices in various regions of the world since 1967. Ridker et al. [2] used the Hedonic Price Model to analyze the effects of air pollution on the prices in the housing market of Saint Louis (MO, USA) for the first time in 1967. Since then, many scholars have contributed to the research on the relationship between air quality and housing prices. Many scholars also carried out extensive analyses on the relationship between air quality and housing prices, based on real estate market transaction data in the US and Europe (Wieand [3], Smith et al. [4], Harrison et al. [5], Nelson [6], Brown et al. [7], Murdoch et al. [8], Chattopadhyay [9], and Zabel et al. [10]). Boyle and Kiel [11] undertook a review on this literature and found that, since the 21st century, with further deterioration of the environment and at the same time improvement of health awareness among the general population, more and more research has focused on the relationship between air quality and housing prices.

By reviewing both related literature and the research on the impact of air quality on housing prices, we can also analyze people’s willingness to pay for a reduction in air pollution and the effect of this preference on housing prices. Bayer et al. [12] calculated that PM_10_ (the particles are smaller than 10 micrometers in size) had a significantly negative effect on local housing prices based on real estate data in some urban areas in the United States from 1990–2000. This research showed that the amount people were willing to pay for a reduction of 1 μg/m^3^ of PM_10_ was $U.S. 53.4 to $U.S. 89.37, which was approximately equal to 0.7% of the local average housing price. In the empirical analysis of the impact of air pollution on local housing rental prices in Jakarta (Indonesia), Yusuf et al. [13] came to the conclusion that suspended particulate matter, SO_2_, and CO all had negative connections with local housing rental prices, and the per family value for a decrease of 1 μg/m^3^ of SO_2_ in Jakarta ranged from $U.S. 28 to $U.S. 85, equivalent to 6.8% of the local average housing rental price. Bajari et al. [14] found that PM_10_ had a negative impact on the values of local housing according to the transaction data from six counties and cities in California’s Bay Area in the United States from 1990–2006, and the amount that local residents were willing to pay for a decrease of 1 μg/m^3^ of PM_10_ ranged from $U.S. 94 to $U.S. 104, roughly equal to 1% of the local average housing value. Chen et al. [15] took China’s city of Qingdao as an example, adopting the Hedonic Price Model to estimate people’s marginal willingness to improve the air quality in residential areas, and concluded that high-income groups were more willing to pay for clean air than low-income groups even when other consumer differences were taken into account. Research conducted by Zhang et al. [16] showed that, based on the data of the commodity housing market of 288 cities in China, with an annual average concentration of PM_10_ decreasing by 1 μg/m^3^, the residents were willing to pay 35.91 yuan more per square meter for housing, which is equal to 0.9% of the average commodity housing price in the same period. Zhang et al. [17] calculated that people on the average were willing to pay ¥258 per year per person for a 1% reduction in PM_2.5_. Ligus [18] attempted to estimate how much Polish citizens would be willing to pay for clean air by applying the Contingent Valuation Method (CVM). Carriazo et al. [19] applied a Second Stage Hedonic Pricing Model by defining intra-urban housing sub-markets. They discovered a negative relationship between PM_10_ concentration and rental prices, which predicted that an increase of 1 µg/m^3^ is accompanied by a monthly average rent reduction of 0.61 percent for apartments, of 2.43 percent for condominiums, and of 2.17 percent for houses. Hitaj et al. [20] investigated the relationship between selling prices of apartments and ambient ozone in Los Angeles through Hedonic Price Model. The estimates of renters’ household annual marginal willingness to pay for a 1% reduction in ambient ozone pollution ranged from $U.S. 14 to $U.S. 52 in constant dollars, which were somewhat smaller than current marginal willingness to pay.

In terms of study methods for this topic, the current mainstream is the combination of spatial econometric models and the Hedonic Price Model, which together fully consider the correlation of housing prices in the study space and subtly avoids the deviation of the estimation results that would occur in the traditional Hedonic Price Model. Five different spatial econometric models, which are able to explicitly take into account spatial effects, have been identified in the literature for cross-section studies. These models are: Spatial Lag Model (SLM), Spatial Error Model (SEM), Spatial Durbin Model (SDM), Geographically Weighted Regression (GWR) and Quantile Regression Models (QRM). The SLM are used to reflect the effect of spatial units on other near units in the whole region, in which spatial lag in a dependent variable is taken into account [21]. In addition, the independent error term may influence on the spatial spillover effects that exist between geographic units. The SEM can solve the problem of spatial autocorrelation with independent error term [22]. The SDM can examine the influence of spatial lag in a dependent variable and spatial error in independent variables [23]. The GWR are employed to analyze the spatial heterogeneity and to measure complex local variation of regression parameters [24]. The QRM can allow parameters to change according to the quantile of dependent variable and use instrument to deal with endogeneity [25]. Anselin et al. [26] combined the Spatial Error Correction Model and Hedonic Price Model to estimate the impacts of air quality on local housing prices of the California housing market in 2000. The research results showed that ozone and air suspended particles were responsible for significantly negative impacts on housing prices, while air quality enhancement in some places in California had significantly increased housing prices. Daniel [27] constructed the Spatial Atmospheric Diffusion Model by using the Spatial Hedonic Price Method, based on the monitoring data of Los Angeles in 1997–2005. This research showed that under the same conditions if nitrogen oxide emissions increased by 1 μg/m^3^, housing values would drop by 0.7%. Tian et al. [28] studied the relationship between transportation infrastructure and housing prices in Saline Lake County of the United States by the methods of Ordinary Least Squares (OLS), Spatial Lag Regression (SLR), and Hierarchical Linear Modeling (HLM). They found that the negative impacts (traffic noise and air pollution) of transportation systems on single-family housing prices were greater than the positive impact (accessibility). Han Li et al. [29] examined consumers’ underlying preferences for various amenities and accessibility factors in Salt Lake County via three models, with a particular focus on air pollutions and forest coverage. Results from three models showed that air pollutions had a significant and detrimental influence on housing values. Neelawala et al. [30] conducted the Ordinary Least Squares (OLS), Spatial Lag Regression (SLR), and Spatial Error Regression (SER) to examine the impact of mining- and smelting-related pollution on nearby property prices with the data of Mount Isa city in Australia, which represented that the marginal willingness to pay to be farther from the pollution was AUS $13,947 per kilometer within the radius of 4 km. To further evaluate how air pollution impact housing prices, Quantile Regression Models (QRM) are used commonly. Chasco et al. [31] used Quantile Regression Models (QRM) with a sample of 5080 houses in the city of Madrid (Spain) and concluded that air pollution had clear significant influence only in the wealthier neighborhoods. And in 2015, Chasco et al. [32] applied Quantile Regression Models (QRM) to estimate the willingness to pay for less air pollution with the data of Madrid and found that implicit prices for good air quality vary greatly in the housing markets, which were mainly caused by perceived intensity of pollution, accessibility to jobs and leisure, and some socio-economic characteristics of the population.

In considering the above literature review, air pollution does affect housing prices, but there are some shortcomings in the existing research. Firstly, the research on the impacts of air quality on housing prices in many cities is gradually developed and completed, but no examination of the relationship between air quality and housing prices with Chengdu’s data has been done. Secondly, the variable of single-family housing prices is regarded as dependent variable in most literature, and it lacks the study of viewing residential communities as the research objects. Thirdly, the existing literature pays much attention to the impacts of air quality on housing selling prices while ignoring the impacts of air quality on housing rental prices, and there is also lack of comparative studies of the impact of air quality on both selling prices and rental prices, especially in China Housing market. Based on the above literature reviews, we use Ordinary Least Square (OLS), Spatial Error Model (SEM) and Spatial Lag Model (SLM) to study the impacts of haze on housing selling prices and housing rental prices, taking 1431 residential communities in 20 districts of Chengdu as the research objects. Furthermore, we also apply Quantile Regression Model (QRM) to explain the heterogeneous and dynamic effects of the haze on house prices at different quantiles in Chengdu’s housing market, one representative Chinese city.

## 3. Materials and Methods 

### 3.1. Study Area

**Figure 1:**
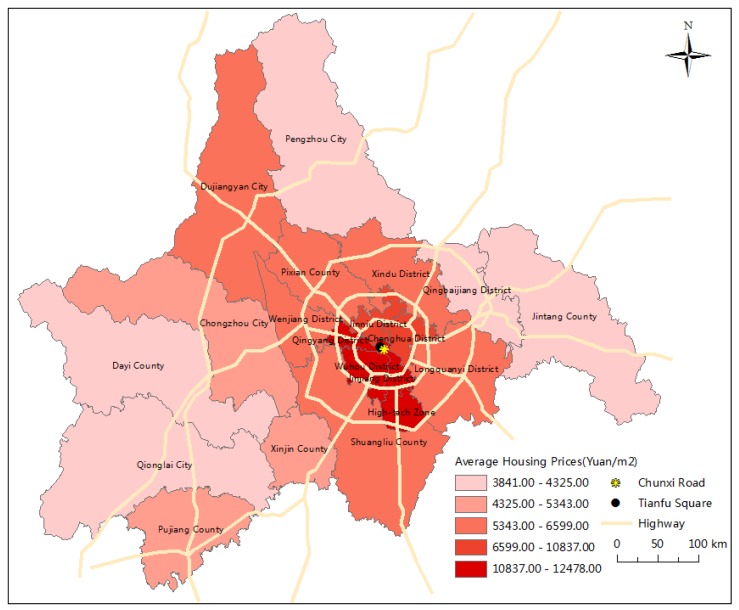
The average housing price pattern in 20 districts of Chengdu in 2016. Data source: Chengdu ReallyDT Platform (www.reallydt.com).

Chengdu is located in the west of the Sichuan basin in China. It is considered a science and technology, commerce, financial, transportation, and communication hub in the southwest of China, and has also always enjoyed the reputation of being “The Land of Abundance”. In 2016, the total Gross Domestic Product (GDP) in Chengdu was 1217.02 billion Yuan, and the contribution of the tertiary industry to this figure was as high as 53.11% [33]. In recent years, Chengdu has actively promoted the development of the real estate industry, and the importance of real estate to the city’s economy has gradually increased. According to data released by the Chengdu Municipal Bureau of Statistics, the proportion of real estate investment to GDP in Chengdu in 2016 was 21.68%, higher than the average of 13.79% in the same period in China as a whole [33]. The average housing price pattern in 20 districts of Chengdu in 2016 is shown in Figure 1; these range from 3841 Yuan/m^2^ to 12,478 Yuan/m^2^. Housing prices in Chengdu were found to vary greatly between the downtown area and its surrounding suburbs. The average housing prices of the downtown area were remarkably higher than those of suburbs.

In line with the development of the real estate industry, the attractive leisure environment has always been a unique advantage of Chengdu in attracting both local and foreign residents to purchase houses, which makes the environmental factors—including air quality—affect the local housing prices significantly. However, with the development of the local economy, Chengdu air quality has shown a trend of deterioration, which means that haze has become an increasingly serious issue. According to the 2016 “*Air Quality Assessment Report (II): Statistical Analysis of Air Pollution in Five Cities of China*”, although the “heavily polluted” air quality rate of Chengdu was not as serious as that of Beijing, the “excellent and good” air quality rate of Chengdu, which was only 12%, was the lowest in the five measured cities [34]. Chengdu’s air quality level was “moderate pollution” or “slight pollution” for most of the year. Understandably, this has heightened local people’s concerns about haze.

### 3.2. Methods

#### 3.2.1. Spatial Autocorrelation

Spatial autocorrelation analysis is used to study the spatial attribution of non-spatial attribute information; that is, to test whether there is a cluster of phenomena in space or not. In general, the commonly used statistics are Moran’s *I* index (Moran, [35]) and Geary’s C index (Geary, [36]). In this paper, both Moran’s *I* index and Geary’s *C* index are adopted to examine the spatial attribution of the selling and rental prices of houses. The computational formulas are as follows:

1. Moran’s *I* Index

(1) $$I=\frac{N\sum_{i}\sum_{j}\omega_{ij}\left(x_{i}-\bar{x}\right)\left(x_{j}-\bar{x}\right)}{\left(\sum_{i}\sum_{j}\omega_{ij}\right)\sum_{i}{\left(x_{i}-\bar{x}\right)}^{2}}$$

where N is the number of evaluation units in the study area, $\omega_{ij}$ is the spatial weight, $x_{i}$ and $x_{j}$ are the respective element attribute values of the evaluation units, and $\bar{x}$ is the average element attribute value of the evaluation units.

The Moran’s *I* index has range of –1 ≤ *I* ≤ 1, where –1 indicates a strong negative spatial autocorrelation, 0 indicates a random distribution, and +1 indicates a strong positive spatial autocorrelation.

2. Geary’s *C* Index
  (2) $$C=\frac{\left(N-1\right)\sum_{i}\sum_{j}\omega_{ij}{\left(x_{i}-x_{j}\right)}^{2}}{2\left(\sum_{i}\sum_{j}\omega_{ij}\right)\sum_{i}{\left(x_{i}-\bar{x}\right)}^{2}}$$

In Equation (2), the meanings of each variable are the same as those in Equation (1). The range of Geary’s *C* index is between 0 and 2. Positive spatial autocorrelation is indicated by values between 0 and 1, random distribution is indicated by a value equal to 1, and negative spatial autocorrelation is found for values between 1 and 2. 

Moran’s *I* index and Geary’s *C* index are similar, but not identical. When calculating Moran’s *I* index the cross product of the median deviation is used, whereas when calculating Geary’s *C* index the deviation between observations is emphasized. As for the statistical test, both Moran’s *I* index and Geary’s *C* index use the Z statistical test of normal distribution. If the Z value is greater than 1.960 of the critical value, the statistical significance is 5%. If the Z value is greater than the critical value 2.576, the statistical significance is at the 1% level.

#### 3.2.2. Hedonic Price Model Modified by the Spatial Regressions

The Hedonic Price Model holds that heterogeneous commodities (real estate goods with typical heterogeneity) are the collection of their intrinsic attributes, and their prices can be expressed by the value of each attribute’s evaluation. The core of the model is to evaluate the implicit price of each attribute by fitting the market transaction data and to establish the function model of the relationship between the values and all attributes, in order to reveal the influences of the attributes on the values. These attributes are divided into three categories: structure characteristics, neighborhood characteristics, and location characteristics. However, the traditional Hedonic Price Model does not explicitly take into account spatial effects in identifying the determinants of house prices, which has certain limitations. With the development of spatial data analysis, there are two main kinds of spatial econometric models considering spatial heterogeneity and spatial dependence. The first one is the Spatial Error Model (SEM). The SEM, which is regarded as the error spatial autocorrelation, reflects that regional spillover is the role of random out. It is applicable to the spatial autocorrelation analysis of the interregional interaction due to its relative position. The second one is the Spatial Lag Model (SLM). The SLM emphasizes the effect of spatial lag in a dependent variable, in which the weighted sum of nearby observations enters as a new independent variable-spatial lag term. Of course, there are Spatial Durbin Model (SDM) and Geographically Weighted Regression (GWR) in the spatial regression analysis. Although the SDM can examine the effects of spatial lag in a dependent variable and spatial error in independent variables, it is mostly used for panel data analysis. And the GWR is one of spatially varying-coefficient regression Models, which are mainly employed to analyze the spatial heterogeneity. Therefore, based on a previous study by Liu and Sun [37], this paper analyses the sample data of 20 districts in Chengdu by using the Hedonic Price Model modified by the spatial errors and the spatial lag in order to fully consider the spatial autocorrelation of housing prices and avoid the deviation caused by the traditional “Hedonic Price Model”. The three forms of the models are as follows:

1. Linear Form

The linear form of SEM is expressed as:

(3) $$P=\beta_{0}+\overset{m}{\underset{K=1}{\sum }}\beta_{K}X_{K}+\mu Z_{I}+\lambda W_{\upsilon }+\varepsilon$$

where *P* is the housing price, $\beta_{0}$ is the constant, $X_{K}$ is the *K* characteristic variable (*K* = 1, 2, …, *m*), m is the number of independent variables except air quality variable, $Z_{I}$ is air quality variable, $\beta_{K}$ and μ are coefficients to be estimated, λ is the spatial error regression coefficient, ε is a random disturbance term, $W_{\upsilon }$ is a proximity-weighted error term, W is the spatial weight matrix; that is, $W=\left[\begin{array}{ccc} W_{11} & \cdots & W_{1K}\\ \vdots & \ddots & \vdots \\ W_{K1} & \cdots & W_{KK} \end{array}\right]$(*K* = 1, 2, …, *m*). As for types of spatial weights matrices, we adopt a contiguity-based spatial weights matrix. If two polygons are contiguous, they are viewed as neighbors. And three basic types of contiguity are rook contiguity (e.g., two polygons share a common border), bishop contiguity (e.g., two polygons share a common vertex) and queen contiguity (e.g., two polygons share either a common border or a common vertex). A contiguity-based spatial weights matrix is typically specified as:

(4) $$W_{ij}=\{\begin{array}{c} 1,\\ 0, \end{array}\begin{array}{c} \mathrm{if}\;i\;\mathrm{and}\;j\;\mathrm{are}\;\mathrm{contiguous},\\ \mathrm{otherwise} \end{array}$$

In our samples, the residential community usually has a close relationship with the surrounding environment. It’s assumed that if distances between two residential communities are within 1 km, they are contiguous. And the queen contiguity is selected to construct a spatial weight matrix.

The linear form of SLM is expressed as:

(5) $$P=\rho W_{P}+\beta_{0}+\overset{m}{\underset{K=1}{\sum }}\beta_{K}X_{K}+\mu Z_{I}+\varepsilon$$

In Equation (5), *ρ* is the spatial lag regression coefficient, $W_{P}$ is the spatially lagged dependent variable, and the meanings of other variables are the same as those in Equation (3).

2. Semi-Logarithmic Form

The semi-logarithmic form of SEM is written as:

(6) $$\ln P=\beta_{0}+\overset{m}{\underset{K=1}{\sum }}\beta_{K}X_{K}+\mu Z_{I}+\lambda W_{\upsilon }+\varepsilon$$

In Equation (6), the meanings of each variable are the same as those in Equation (3).

The semi-logarithmic form of SLM is written as:

(7) $$\ln P=\rho W_{\ln P}+\beta_{0}+\overset{m}{\underset{K=1}{\sum }}\beta_{K}X_{K}+\mu Z_{I}+\varepsilon$$

In Equation (7), the meanings of each variable are the same as those in Equation (5).

3. Logarithmic Form

The logarithmic form of SEM is specified as:

(8) $$\ln P=\beta_{0}+\overset{m}{\underset{K=1}{\sum }}\beta_{K}\ln X_{K}+\mu \ln Z_{I}+\lambda W_{\upsilon }+\varepsilon$$

In Equation (8), the meanings of each variable are the same as those in Equation (3).

The logarithmic form of SLM is specified as:

(9) $$\ln P=\rho W_{\ln P}+\beta_{0}+\overset{m}{\underset{K=1}{\sum }}\beta_{K}\ln X_{K}+\mu \ln Z_{I}+\varepsilon$$

In Equation (9), the meanings of each variable are the same as those in Equation (5).

#### 3.2.3. Quantile Regression Model

The Quantile Regression Model (QRM) estimates a conditional quantile function, which means that it can create different regression lines for different quantiles of the dependent variable. The quantile estimators are less influenced by the outliers in the database and more robust. In fact, the real estate has typical heterogeneity, and the impacts of haze on the quantiles of housing prices are different. In OLS, SEM or SLM, the estimation results focus on the average marginal effect of haze on housing prices, which are easily affected by the degree of heterogeneity in the database. Therefore, by using the QRM, we can examine the influences of haze on different quantiles of housing prices, establish the relationship between housing prices and consumers’ willingness to pay for good air quality, and then analyze the connection between consumers’ willingness to pay and their incomes. Formally, the QRM can be formalized as follows:

(10) $$Q_{\tau }\left(P|X\right)=\beta_{0}\left(\tau \right)+\overset{m}{\underset{K=1}{\sum }}\beta_{K}\left(\tau \right)X_{K}+\beta_{Z}\left(\tau \right)X_{Z}+\varepsilon \left(\tau \right)$$

where τ is the probability level, ranging from 0 to 1; $Q_{\tau }$ is the value at quantile τ; P is the housing prices; $\beta_{0}$ is the constant; $\beta_{0}\left(\tau \right)$ is the constant at quantile τ; $X_{K}$ is the *K* characteristic variable (*K* = 1, 2,…, *m*); m is the number of independent variables except air quality variable; $X_{Z}$ is air quality variable; $\beta_{K}$ and $\beta_{Z}$ are the vector of regression parameters; and ε(τ) is a random disturbance term.

In the QRM, a linear programming method (LPM) is generally used to estimate the minimum weighted absolute deviation, then the regression coefficient of the explanatory variable is calculated. Taking the variable of air quality as an example, the estimation of $\beta_{Z}\left(\tau \right)$ is obtained as follows:

(11) $$\beta_{Z}\left(\tau \right)=argmin\left\{\overset{n}{\underset{i:P_{\left(i\right)}\geq Q_{\tau }{\left(P|X\right)}_{\left(i\right)}}{\sum }}\tau \left|P_{\left(i\right)}-Q_{\tau }{\left(P|X\right)}_{\left(i\right)}\right|+\overset{n}{\underset{i:P_{\left(i\right)}<Q_{\tau }{\left(P|X\right)}_{\left(i\right)}}{\sum }}\left(1-\tau \right)\left|P_{\left(i\right)}-Q_{\tau }{\left(P|X\right)}_{\left(i\right)}\right|\right\}$$

where *argmin*{·} is the value of $\beta_{Z}$ when the function takes the minimum value, *n* is the number of samples (i = 1, 2, … *n*).

### 3.3. Data Variables

There are two ways to select variables when researching the influence of haze on housing selling prices and rental prices. One is to find out the existing research results that affect housing prices through a literature review (the results of which are shown in Table 1). The other is to understand the concepts of healthy habitancy and the basic situation of purchasing houses with good air quality through social research methods, such as symposiums and field surveys.

The residence communities of Chengdu are taken as the analysis units, and the average selling prices and rental prices of residential housing in 2016 are taken as the dependent variables; that is, the unit selling price (Yuan/m^2^) and unit monthly rental price (Yuan/month/m^2^) of the residence community. Based on the existing literatures combined with the specific characteristics of the Chengdu residence communities, the availability of data and the research focus, this paper selects residential characteristic variables including structure characteristics, neighborhood characteristics, location characteristics, and air quality characteristics, for a total of 16 variables. All of the variables are shown in Table 2.

The structure characteristics primarily include three variables, among them the age of the building and the total floor number. It is expected that the older the building is, the lower the selling price or rental price will be. On the other hand, the more parking spaces there are, the higher the selling price or rental price will be. The total number of floors in the residential communities has an unknown influence on selling and rental prices.

The neighborhood characteristics include seven variables: volume rate, green rate, surrounding environment, property management, cultural and sports facilities, living facilities, and educational facilities. It is expected that all the variables have positive effects on housing selling prices and housing rental prices, except for the variables of volume rate and surrounding environment. To put it in another way, the lower the volume rate is, the higher the selling prices and rental prices will be. Higher the green rate; closer to the park; higher property management service all have significant effects on housing prices. Finally, the more complete the cultural facilities, living facilities, and educational facilities are, the higher the selling prices and rental prices will be.

The location characteristics include five variables: bus condition, subway condition, shopping condition, distance to Tianfu Square, and distance to Chunxi Road. It is expected all five of these variables will have a negative influence on housing selling prices and housing rental prices. In other words, the more convenient the traffic is, the higher the selling prices and rental prices will be. The handier the shopping conditions are, the higher the selling prices and rental prices will be. Finally, the shorter the distance to the city center is, the higher the selling prices and rental prices will be.

The air quality characteristic only contains one variable: the air quality index. It is expected that the poorer the air quality is, the lower the housing selling prices and rental prices will be.

The quantification of the above 16 variables can be divided into four categories. The first way to divide the variables is to use the actual values of the residential characteristic variables, or the simply-converted values. There are 11 variables in this category, such as the housing age and total number of floors. The second way is to adopt the dummy variables for quantification, and the parking coefficient variable belongs to this category. The third way is to utilize a 5 point Likert scale which only measures the property management variable. The fourth way is to accept only the most comprehensive indicators, consisting of the cultural and sports facilities, living facilities, and education facilities.

### 3.4. Data Sources

**Figure 2:**
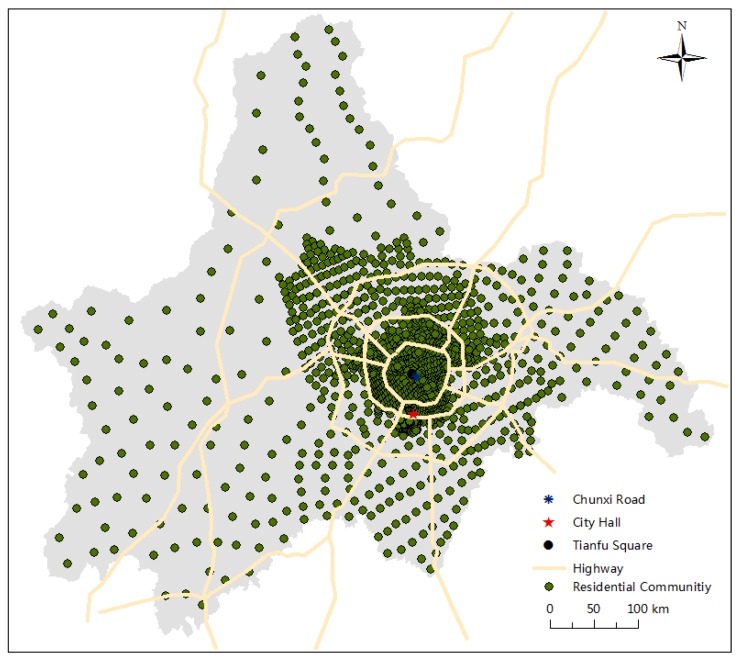
The distribution of residential communities. Data source: Chengdu ReallyDT Platform (www.reallydt.com).

The data in this article comes from three databases. The first data source is Chengdu ReallyDT Platform (www.reallydt.com), a real estate intermediary service company in Chengdu with a history of nine years. Its database stores real estate macro data of more than 40 cities in China and real estate detailed data of 10 key cities, which is related to policies, economy, land, supply, transaction, inventory, advertising, marketing campaigns, products, customers and so on. And it has already been one of the most professional and authoritative real estate service companies in western China. In our research, we selected a total of 1431 samples by eliminating missing values. It accounts for approximately 95.40% of the total transaction volume of Chengdu’s residential communities in 2016. The distribution of residential communities is shown in Figure 2. Eventually, cross-section data in Chengdu in 2016 include housing selling prices, housing rental prices, residential location, total number of floors, age of building, volume rate, green rate, and property management.

The second source of data is Google Maps. According to the location of the residence community and other information, this article collects the straight distances from the residence community to the downtown of Chengdu (Tianfu Square) and the traditional commercial center (Chunxi Road), gathers the linear distance from the residence community to the nearest park, the nearest bus station, the nearest subway station and the nearest shopping center, and organizes the number of schools, hospitals, gyms, and so on within 1 km of the residential area, through Google Maps.

The third dataset was from the “*Chengdu Air Quality Report in 2016*”, provided by the Chengdu Environmental Protection Bureau. This report published the monthly air quality index, air quality class, and primary pollutants in Chengdu from 2012. The air quality index is non-dimension index that describes the comprehensive status of urban air quality. It considers the pollution levels of various pollutants, including sulfur dioxide (SO_2_), nitrogen dioxide (NO_2_), nitric oxide (NO), ozone (O_3_), particulate matter (PM_10_) and fine particulate matter (PM_2.5_). The greater the air quality index is, the heavier the integrated pollution is. According to the index, China’s relevant departments release severely polluted weather warnings. And the index is also a vital basis for school closure. The air quality index values of 20 districts in Chengdu were obtained from the report [38]. All of the observations are derived from eight national air quality monitoring sites in Chengdu, allowing the spatial line distance between the residential quarters and all observing sites to be obtained. This article refers to the “inverse distance weighted interpolation” method advocated by Luechinger [39] and Chen et al. [40] to calculate the air quality index between each cell. The computational formula is as follows:

(12) $$AQI=\frac{\sum_{m=1}^{i}AQI_{m}\cdot e^{-3d_{m}}}{\sum_{m=1}^{i}e^{-3d_{m}}}$$

where AQI is the air quality index, m is the air quality monitoring site, i=8, $d_{m}$ is the straight-line distance from the cell to the monitoring site m, and $AQI_{m}$ is the air quality index at the monitoring site m. According to the minimized root mean square prediction error (RMSPE), the optimal parameter value is determined as 3, which is same as related settings used by Luechinger [39] and Chen et al. [40]. We take $e^{-3d_{m}}$ as distance weight value. It can be more stable based on the distance of the output point to control the influence of the known points to the interpolation, so that the whole space surface is closer to the authenticity.

Before the model is estimated the data is preprocessed to eliminate the outliers, leaving the number of effective samples at 1431, as shown in Table 3.

## 4. Results

**Figure 3:**
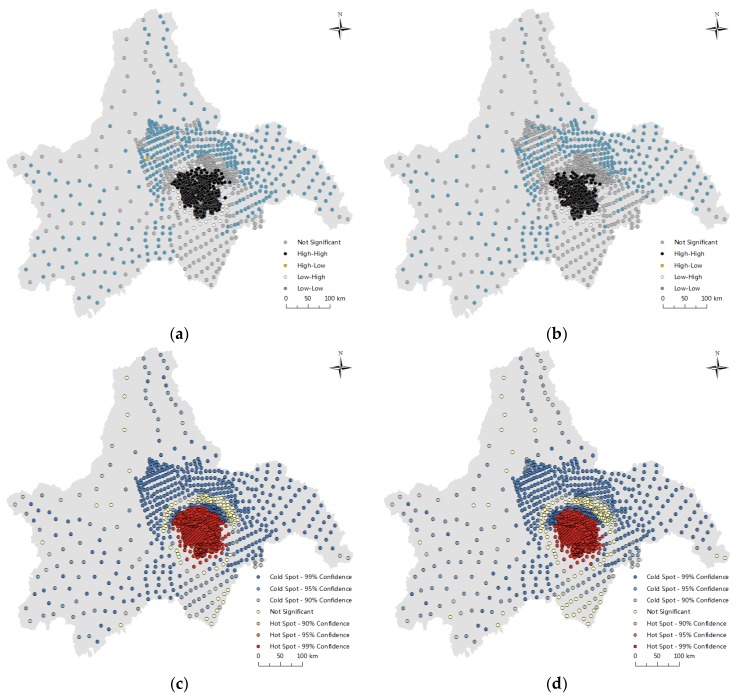
The spatial distribution of housing selling prices and housing rental prices in Chengdu in 2016. Note: (**a**) The spatial clustering of housing selling prices (high-high, low-low); (**b**) The spatial clustering of housing rental prices; (**c**) The distribution of hot spots of housing selling prices (confidence levels were 90%, 95% and 99% respectively); (**d**) The distribution of hot spots of housing rental prices.

Firstly, to examine whether the observed values of housing selling prices and housing rental prices are related to the spatial location or not, this paper carried out a spatial autocorrelation analysis of housing selling prices and housing rental prices through the Arcgis10.2 software (ESRI, Redlands, CA, USA). The spatial distribution of housing selling prices and housing rental prices are displayed in Figure 3. The Moran’s *I* index and Geary’s *C* index values are shown in Table 4.

Figure 3 indicates that housing selling prices and housing rental prices have clustering distribution in space with high-high (hot spot) and low-low (cold point). So spatial autocorrelation should be considered in data analysis. As shown in Table 4, the Moran’s *I* index value of the housing selling prices is 0.4718, and its Z value is 140.0471; the Geary’s *C* index value of housing selling prices is 0.0004 (from 0–1), and its Z value is 30.9766. These two statistical values indicate that there is a positive spatial correlation of housing selling prices in space at the significance level of 1%. In addition, the Moran’s *I* index value of housing rental prices is 0.4870, and its Z value is 144.5737; the Geary’s *C* index value of housing rental prices is 0.0004 (from 0–1), and its Z value is 30.3103. These two values demonstrate that there is a positive spatial correlation of housing rental prices in space at the significance level 1%. To summarize, both housing selling prices and housing rental prices have strong spatial clustering characteristics; that is, positive spatial autocorrelation. Therefore, this paper further adopts the Hedonic Price Model modified by the spatial errors and the spatial lag to simulate the sample data.

### 4.1. The Impact of Haze on Housing Selling Prices

In order to fully reflect the spatial autocorrelation and agglomeration effect of the housing selling prices the OLS regression model, the Spatial Error Model (SEM) and the Spatial Lag Model (SLM) were constructed in this section, in which the variable of housing selling prices is regarded as the dependent variable, while the 16 characteristic variables were used as independent variables. In determining the best form of the Hedonic Price Model corrected by the spatial error and the spatial lag, three kinds of forms were obtained by the GeoDa software (The University of Chicago, Chicago, IL, USA). Measures of goodness-of-fit such as R-squared, Log likelihood, Akaike Information Criterion (AIC) and Schwarz Criterion (SC) were reported for each model. The regression result shows that the logarithm form has the best fitting effect of the three forms. Therefore, the logarithmic form was used to analyze the effect of haze on housing selling prices. The regression results are presented in Table 5.

Table 5 shows that the results of the spatial regression analysis are basically consistent with the OLS regression in terms of the regression coefficient and significance of the independent variables. However, the distance to Chunxi Road has significant relationship with housing selling prices in SEM, while in the other two models it is not significant. One possible reason for this could be that the mutual influence of random residuals between different regions leads to different significance levels of this variable. The spatial error λ is obtained through the significance test (*p* = 0.000 < 0.01) and the spatial lag ρ is also obtained through the significance test (*p* = 0.000 < 0.01). The R-squared and Log likelihood are increasing from OLS to spatial regression models, and the AIC and SC are decreasing from OLS to spatial regression models, which mean that it’s essential to replace OLS with spatial regression models, which is consistent with the test of spatial autocorrelation. To identify whether the autocorrelation is the values of the dependent variable or in its errors, a Lagrange Multiplier (LM) test and a Robust Lagrange Multiplier (Robust-LM) test were conducted by the Geoda software. The result of LM test shows that both spatial error and spatial lag are significant at the level of 1%. However, the result of Robust-LM test represents that the autocorrelation in the dependent variable is less strong than that in the errors. Goodness-of-fit statistics such as the R-squared, Log likelihood, AIC and SC can also be used to estimate the fitting degree of regressions. The SEM has the best fitting effect of the three models Therefore, the results of the SEM should be adopted first.

According to the results of the Spatial Error Model, there are 13 significant variables among the 16 variables involved in the regression model. While the variables of shopping malls and distance to Chunxi Road are significant at the 5% level, the rest are significant at 1%. These variables are: the air quality index, parking coefficient, volume rate, green rate, property management, cultural and sports facilities, educational facilities, surrounding environment, bus, subway, and distance to Tianfu Square. At the same time, the air quality index in the model is less than 1% of the significance level, and the coefficient is negative. This hints that the air quality index has significantly negative impacts on housing selling prices. The lower that the air quality index is, the higher the housing selling prices are. That is to say, residents are willing to pay for good air quality, and their “willingness to pay” has already been capitalized into the housing selling prices. The air quality index is non-dimension index that describes the comprehensive status of air quality. The regression coefficient for the air quality index is −0.3967, which implies that a unit of housing selling price falls by 39.67% when the air quality index rises by 1. Actually, in practical applications, the changes of the air quality index of each region in Chengdu are relatively small. According to the previous descriptive analysis of the air quality index, the average air quality index of each region in Chengdu in 2016 varies between 5.06 and 7.09. Therefore, in combination with the actual conditions of Chengdu, the unit of change in the air quality index is set to 0.1. In other words, a unit of housing selling price falls by 3.97% approximately when the air quality index rises by 0.1. In the sample data, the average housing selling price is 7604.42 Yuan/m^2^. In other words, the housing selling price falls by 301.90 Yuan/m^2^ when the air quality index rises by 0.1.

### 4.2. The Impact of Haze on Housing Rental Prices

The empirical analysis of the housing selling market shows that air quality has been partly capitalized into housing prices. Furthermore, the better the air quality is, the higher the capitalization rate is. We queried whether the same conclusion could be drawn for the housing rental market and in this section, the data of the housing rental prices was collected in the same residential communities as the housing selling market. Then, the OLS regression model, the SEM and the SLM were also set up in which the value of the housing rental prices was regarded as the dependent variable, while the 16 characteristic variables were used as independent variables. Measures of goodness-of-fit such as R-squared, Log likelihood, AIC and SC were reported for each model. It was determined that the best fitting effect is the logarithmic form. Therefore, the logarithmic form was used to analyze the effect of haze on housing rental prices. The regression results are shown in Table 6.

Table 6 presents the results of the spatial regression analysis and demonstrates that they are basically consistent with the OLS regression in terms of the regression coefficient and significance of the independent variables. However, the significance of two variables, volume rate and educational facilities in SEM are different from the other two models. The possible reason is same with the Chunxi road estimation in Table 5. To identify whether the autocorrelation is the values of the dependent variable or in its errors, a Lagrange Multiplier (LM) test and a Robust Lagrange Multiplier (Robust-LM) test are conducted by the Geoda software. The result of LM test and Robust-LM test represents that both spatial error and spatial lag are significant at the level of 1%. Goodness-of-fit statistics such as the R-squared, Log likelihood, AIC and SC can also be used to estimate the fitting degree of regressions. The SEM has the best fitting effect of the three models. Therefore, the results of the SEM should be accepted first.

According to the results of the spatial error analysis, among the 16 variables involved in the regression there are eight significant variables in the regression model. Five of those variables are significant at the 1% significance level, including the air quality index, property management, cultural and sports facilities, surrounding environment, and subway variables. Meanwhile, the air quality in the model is less than 1% of the significance level, and the coefficient is negative. This indicates that the negative impact of the air quality index on housing rental prices is notable. The lower the air quality index is, the higher the housing rental prices are. In other words, renters are also willing to pay for good air quality, and part of the cost has been capitalized into housing rental prices. The coefficient of the air quality index is −0.4013. That is to say, when the air quality index rises by 0.1, the unit housing rental price drop by about 4.01% on average. In the sample data, the average rental price of housing is 22.45 Yuan/month/m^2^. In other words, when the air quality index rises by 0.1, the unit housing rental price drop by 0.90 Yuan/month/m^2^. With a house of 25 m^2^ as a sample, the average annual rent is 6735 Yuan. Therefore, when the air quality index rises by 0.1, the annual housing rental price fall by 270.07 Yuan.

### 4.3. Comparison of the Impacts of Haze on Housing Selling Prices and Housing Rental Prices

Based on the previous analyses, the data shown in Table 7 are obtained. As shown in Table 7, haze has negative impacts on housing selling prices and housing rental prices. If the other factors remain unchanged, when the air quality index rises by 0.1, the housing selling prices will drop by 3.97% and the housing rental prices will drop by 4.01%. This shows that residents are willing to pay extra for good air quality, and the impacts of air quality are reflected in the housing prices.

At the same time, housing rental prices are more sensitive to the air quality than housing selling prices, which reflects the differences between China’s housing rental market and housing selling market. Housing selling prices are often affected by policies, investments, and other non-market factors. Additionally, the cost of capital investment in the housing selling market is relatively high, which affects the consideration of other property characteristics of the housing demand group. These combined factors lead to the relatively lower influence of the haze factor on housing selling prices. However, the housing rental market is affected by human factors. The capital investments of its demand group are low and flexible, suggesting that changes in the rental prices are more sensitive. Hence, the impacts of haze on the housing rental prices are more obvious.

### 4.4. Effects of the Haze on House Prices at Different Quantiles

In this section, we utilized Quantile Regression Model (QRM) to explain the heterogeneous and dynamic effects of the haze on house prices at different quantiles in Chengdu’s housing market. In the QRM, the dependent variable is housing selling prices, the 16 characteristic variables are used as independent variables. Measures of goodness-of-fit such as Pseudo R-squared, and Quasi-LR statistic were reported. It was determined that the best fitting effect is the logarithmic form. The regression results are shown in Table 8.

**Figure 4:**
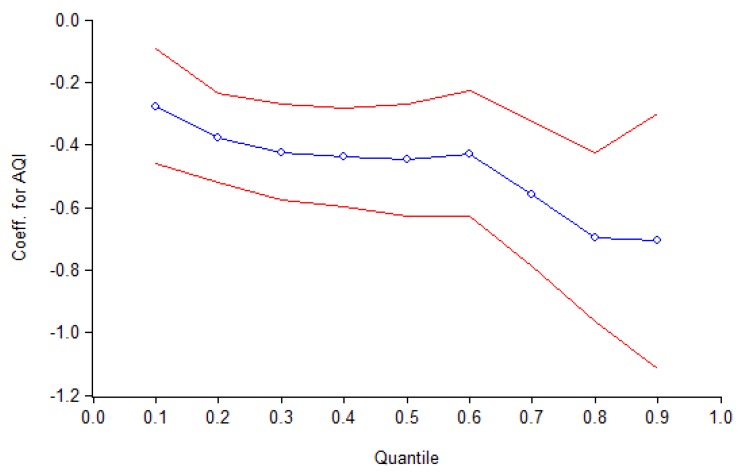
Quantile regression estimates with 95% confidence interval for the impact of haze on housing selling prices. Blue line indicates result from quantile regression. Red lines show 95% confidence interval for quantile regression.

It can be seen from Table 8 that the air quality index is less than 1% in the QRM, and all coefficient estimations are statistically negative. That is to say, the consumers are willing to pay premium for good air quality. From Figure 4, it is found that with the increase of the quantiles, the coefficient of the air quality index is greater, which means the consumers prefer to pay more premium for good air quality. That is, the consumers with different consumption capacity have a different sensitivity to haze. When the quantile is equal to 0.1, the coefficient estimation of air quality on housing selling prices is −0.2765. The consumers’ willingness to pay for good air quality is about 2.77% of the price of housing. When the quantile rises to 0.9, the coefficient is −0.7077. The consumers’ willingness to pay for good air quality is about 7.08% of the price of housing. The coefficient of the latter is 2.56 times that of the former. The difference comes from the different motives of the buyers with different incomes. Buyers who prefer houses with high price, generally belong to the high income and high education class and they pay more attention to healthy living and have lower tolerance for haze pollution, so they would pay a premium for good air quality. For the buyers who prefer houses with lower price, the primary motivation is to meet their housing needs. They are less sensitive to the surrounding haze pollution, hence, they are often reluctant to pay too high prices for improving air quality in their house purchasing behavior.

## 5. Discussion

In order to explore the social effects of haze on housing prices, some interviews and investigations were conducted in relevant individual communities. These were mainly focused on the concept of a healthy life and the situation of purchasing houses with good air quality. 

Firstly, the impact of haze on housing prices suggests the active pursuit of healthy living among residents in the study area. In the interview, people generally expressed that Chengdu’s haze pollution is becoming increasingly serious, and most of the respondents indicated that they will consider the air quality of the living area and the surrounding environment in their purchase of housing, which due to air quality has significant impacts on family living quality and overall well-being. In fact, in the process of China’s urbanization, the construction of ecological civilization has always lagged behind the scale of urban expansion, and urban environmental pollution is consequently increasingly aggravating—especially air pollution. In this “smoggy and foggy” environment, the haze has become a new normal pollution. However, with the promotion of environmental cognition, people will tend to manifest “pro-environment behavior”, and this drives competitive behavior for good air quality housing under a climate of risk aversion. As a result, air quality influences housing prices more significantly.

Secondly, the impacts of haze on housing prices intensifies the “crowding out effect “on talent in urban areas, especially for the elite and affluent class. Typically, the elite and affluent class pay more attention to healthy living and have lower tolerance to haze pollution. Therefore, they are often willing to pay a higher proportion of their incomes to enhance the air quality in their residential area, and generally live in areas with good air quality and higher housing prices. This results in the areas with good air quality driving a “crowding out effect” of talent from other areas with poor air quality. It can be inferred that the current population flow among urban areas is not only dependent on the level of regional economic development and social employment opportunities, but also on the superior environment and air quality in the interior region. This will provide sustainable human capital for economic development, which is conducive to the accumulation of talent. Of course, it also increases the external efficiency of good air quality areas and accelerates the outflow of talent and elements from other areas with high haze pollution, which further widens the gap of regional talents both in quantity and quality. The difference of urban regional economic and population structures between good and poor air quality areas not only forms a region “fence” within cities, but also destroys the stability and harmony between urban inner regions. Eventually, it will affect the long-term and healthy development of the city. The important finding for current local governments from this research is that deterioration of local haze pollution will lead to the loss of talent in certain areas. It is essential for local governments to provide adequate investment into environmental protection, in order to alleviate the deterioration of regional air quality.

## 6. Conclusions

This paper takes Chengdu as an example and analyzes the impacts of haze on housing selling prices and housing rental prices through combining the Spatial Error Model and the Spatial Lag Model with the Hedonic Price Model. The results illustrate that haze has negative impacts on housing selling prices and housing rental prices. Controlling other housing factors, air quality index increases of 0.1 causes a housing selling price drop 3.97%, and rental price drop 4.01%. This demonstrates that residents are willing to pay a premium for good air quality, and the impacts of air quality are embodied in the housing prices. At the same time, housing rental prices are more sensitive to the air quality than housing selling prices, which reflects that China’s rental market responds to the changes of its own market prices more sensitively. There are however certain limitations in this article. For example, we select the queen contiguity to conduct a spatial weights matrix, but do not adopt other forms of weighting matrices to measure. Actually, the results of the spatial regression models might be very sensitive to the ways of constructing the spatial weights matrices, which should be improved in future studies.

## Figures and Tables

**Table 1:** Results from the existing literature.

Types	Number of References	Specific Variables
Structure Characteristics	11	Age of building, total number of floors, land area, construction area, residential usable floor area, number of bedrooms, number of bathrooms, number of fireplaces, whether apartment is located in a gated community, whether there is a courtyard, whether there is a swimming pool
Neighborhood Characteristics	5	School quality, population density in residential area, employment rate in residential area, crime rate in residential area, and payment of real estate tax
Location Characteristics	8	Number of bus stops within half a mile, number of light rail stations within half a mile, distance to the nearest expressway exit, distance to the nearest mall, distance to the nearest hospital, distance to the nearest conservation, distance to the nearest factory, distance to the nearest recreational facility, distance to downtown center, number of subway stations within 300 m, number of parks within 500 m, whether apartment is located in the catchment zone of municipal key elementary or secondary schools
Socioeconomic Characteristics	4	Annual income of the family, non-housing expenditure of the family, rate of unemployment in the residential area, proportion of the white people in the residential area, proportion of the undergraduate and above education in the residential area, sex of the buyers, and marital status of the buyers
Environmental Characteristics	11	Area of green space, green rate, elevation of housing location, slope of housing location, distance to the nearest water source, quality of the nearest water source, traffic noise index, air pollution component index

**Table 2:** Variable definition and expected symbol.

Type	Variable Name	Descriptions	Expected Symbol
Structure Characteristics	Age of building	Housing construction age in the residence community (years).	−
	Total number of floors	Total number of floors in the residence community (levels).	unknown
	Parking coefficient	The total number of parking spaces in the residence community divided by the ratio of the total number of households: if the parking coefficient is ≥1 it is assigned to 1, and if it is less than 1 is assigned to 0.	+
Neighborhood Characteristics	Volume rate	The ratio of total construction area to land area of the residence community.	−
	Green rate	Percentage of green area in the residence community (%).	+
	Surrounding environment	Distance to the nearest park (km).	−
	Property management	The service quality of property management is generally measured by the qualifications of the Property Management Company, which is divided into five grades: great (5 points), good (4 points), general (3 points), bad (2 points), terrible (1 point).	+
	Cultural and sports facilities	Type of cultural and sports facilities within 1 km of the residence community: sports field, gym, badminton court, basketball court, tennis hall, swimming pool, elderly activities room, children’s palace, cinema, and so on (each one gets 1 point, with a total of 5 points).	+
	Living facilities	Types of living facilities within 1 km of the residence community: supermarket, food market, banks, courier points and hospitals (each one gets 1 point, with a total of 5 points).	+
	Educational facilities	Type of educational facilities within 1 km of the residence community: kindergartens, primary schools, junior high schools, high schools and universities (each one gets 1 point, with a total of 5 points).	+
Location Characteristics	Bus	Distance to the nearest bus station (km).	−
	Subway	Distance to the nearest subway station (km).	−
	Shopping mall	Distance to the nearest shopping mall (km).	−
	Distance to Tianfu Square	Distance to Tianfu square (km).	−
	Distance to Chunxi Road	Distance to Chunxi Road (km).	−
Air Quality Characteristic	Air quality index	Air quality index in the residence community.	−

+: positive impact; −: negative impact.

**Table 3:** Descriptive statistical analysis.

Variable Name	Min	Max	Mean	Std. Deviation
Housing selling prices	2245	28,705	7604.42	3113.418
Housing rental prices	6.48	80.00	22.45	9.540
Age of building	0	20	4.63	3.842
Total number of floors	4	55	22.08	9.842
Parking coefficient	0	1	0.42	0.493
Volume rate	0.30	14.49	3.53	1.434
Green rate	10.00	90.00	33.49	9.904
Surrounding environment	0.01	7.70	1.41	1.034
Property management	1	5	3.55	1.002
Cultural and sports facilities	1	5	3.44	1.557
Living facilities	1	5	4.56	0.855
Educational facilities	1	5	3.34	1.142
Bus	0.01	7.70	0.34	0.558
Subway	0.02	67.80	8.15	13.030
Shopping mall	0.00	26.60	1.46	2.037
Distance to Tianfu Square	0.35	78.80	15.92	14.848
Distance to Chunxi Road	0.47	79.80	16.10	14.981
Air quality index	5.06	7.09	6.37	0.452

**Table 4:** Spatial autocorrelation analysis of housing prices (*n* = 1431).

Spatial Clustering Index		Housing Selling Prices	Housing Rental Prices
Moran’s *I* index	Index value	0.4718 ***	0.4870 ***
	Expected value	−0.0007	−0.0007
	Variance	0.00001	0.00001
	Z-value	140.0471	144.5737
Geary’s *C* index	Index value	0.0004 ***	0.0004 ***
	Expected value	0.0003	0.0003
	Variance	0.0000	0.0000
	Z-value	30.9766	30.3103

Note: *** *p* < 0.01.

**Table 5:** OLS regression, Spatial Error Model and Spatial Lag Model results for housing selling prices (*n* = 1431).

Independent Variable	OLS Regression	Spatial Error Model	Spatial Lag Model
Constant	9.8990 *** (0.1645)	9.8160 *** (0.2165)	9.6737 *** (0.1812)
Age of building	−0.0137 (0.0107)	−0.0103 (0.0104)	−0.0138 (0.0106)
Total number of floors	−0.0164 (0.0138)	−0.0014 (0.0132)	−0.0129 (0.0137)
Parking coefficient	0.0502 *** (0.0159)	0.0408 *** (0.0151)	0.0485 *** (0.0151)
Volume rate	−0.0780 *** (0.0162)	−0.0867 *** (0.0154)	−0.0780 *** (0.0161)
Green rate	0.0919 *** (0.0182)	0.0844 *** (0.0173)	0.0910 *** (0.0181)
Surrounding environment	−0.0423 *** (0.0072)	−0.0417 *** (0.0069)	−0.0413 *** (0.0071)
Property management	0.2833 *** (0.0210)	0.2600 *** (0.0200)	0.2779 *** (0.0208)
Cultural and sports facilities	0.0589 *** (0.0111)	0.0478 *** (0.0107)	0.0601 *** (0.0110)
Living facilities	0.0065 (0.0224)	0.0170 (0.0218)	0.0075 (0.0222)
Educational facilities	0.0625 *** (0.0137)	0.0392 *** (0.0132)	0.0636 *** (0.0136)
Bus	−0.0329 *** (0.0065)	−0.0251 *** (0.0066)	−0.0343 *** (0.0065)
Subway	−0.0671 *** (0.0061)	−0.0593 *** (0.0062)	−0.0657 *** (0.0061)
Shopping mall	−0.0129 ** (0.0065)	−0.0123 ** (0.0062)	−0.0127 ** (0.0064)
Distance to Tianfu Square	−0.2250 *** (0.0314)	−0.1806 *** (0.0333)	−0.2223 *** (0.0311)
Distance to Chunxi Road	−0.0447 (0.0292)	−0.0738 ** (0.0321)	−0.0431 (0.0290)
Air quality index	−0.4286 *** (0.0769)	−0.3967 *** (0.1083)	−0.4039 *** (0.0767)
Spatial error (λ)	—	0.4796 *** (0.0437)	—
Spatial lag (ρ)	—	—	0.0192 *** (0.0068)
R-squared	0.7789	0.7962	0.7802
Log likelihood	362.7860	403.6691	366.8450
Akaike Information Criterion (AIC)	−691.5710	−773.3380	−697.6890
Schwarz Criterion (SC)	−602.0470	−683.8139	−602.8990
Lagrange Multiplier (LM)	—	94.1331 ***	8.1721 ***
Robust Lagrange Multiplier (Robust-LM)	—	89.2213 ***	3.2603 *

Note: * *p* < 0.1, ** *p* < 0.05, *** *p* < 0.01, the value in parentheses is the standard error of the variable.

**Table 6:** OLS regression, Spatial Error Model and Spatial Lag Model results for housing rental prices (*n* = 1431).

Independent Variable	OLS Regression	Spatial Error Model	Spatial Lag Model
Constant	4.0084 *** (0.2164)	3.8038 *** (0.2968)	3.0971 *** (0.2303)
Age of building	−0.0166 (0.0141)	−0.0041 (0.0135)	−0.0112 (0.0135)
Total number of floors	−0.0085 (0.0182)	0.0105 (0.0171)	0.0075 (0.0175)
Parking coefficient	0.0458 ** (0.0209)	0.0366 * (0.0195)	0.0396 ** (0.0201)
Volume rate	−0.0278 (0.0213)	−0.0365 * (0.0199)	−0.0278 (0.0206)
Green rate	0.0171 (0.0240)	0.0270 (0.0224)	0.0148( 0.0231)
Surrounding environment	−0.0370 *** (0.0094)	−0.0332 *** (0.0090)	−0.0316 *** (0.0091)
Property management	0.2588 *** (0.0276)	0.2144 *** (0.0259)	0.2281 *** (0.0267)
Cultural and sports facilities	0.0715 *** (0.0145)	0.0450 *** (0.0139)	0.0689 *** (0.0140)
Living facilities	0.0298 (0.0295)	0.0049 (0.0283)	0.0254 (0.0284)
Educational facilities	0.0417 ** (0.0180)	0.0132 (0.0172)	0.0383 ** (0.0174)
Bus	−0.0009 (0.0086)	−0.0004 (0.0086)	−0.0064 (0.0083)
Subway	−0.1001 *** (0.0080)	−0.0824 *** (0.0081)	−0.0862 *** (0.0078)
Shopping mall	−0.0090 (0.0085)	−0.0081 (0.0081)	−0.0100 (0.0082)
Distance to Tianfu Square	−0.1180 *** (0.0413)	−0.0550 * (0.0437)	−0.0883 * (0.0399)
Distance to Chunxi Road	−0.0248 (0.0384)	−0.0811 (0.0424)	−0.0227 (0.0370)
Air quality index	−0.4901 *** (0.1011)	−0.4013 *** (0.1497)	−0.3557 *** (0.0983)
Spatial error (λ)	—	0.5439 *** (0.0403)	—
Spatial lag (ρ)	—	—	0.1931 *** (0.0215)
R-squared	0.6112	0.6512	0.6346
Log likelihood	−29.0214	25.6255	12.9497
Akaike Information Criterion (AIC)	92.0428	−17.2510	10.1006
Schwarz Criterion (SC)	181.5670	72.2732	104.8910
Lagrange Multiplier (LM)	—	120.8174 ***	89.8179 ***
Robust Lagrange Multiplier (Robust-LM)	—	65.5729 ***	34.5734 ***

Note: * *p* < 0.1, ** *p* < 0.05, *** *p* < 0.01, the value in parentheses is the standard error of the variable.

**Table 7:** Comparison of the effects of haze on housing selling prices and housing rental prices.

Dependent Variable	Regression Coefficient	Average Price/Rent	Marginal Price/Rent
Housing selling prices	−0.3967	7604.42 Yuan/m^2^	301.90 Yuan/m^2^
Housing rental prices	−0.4013	22.45 Yuan/month/m^2^	0.90 Yuan/month/m^2^

**Table 8:** The quantile regression results for housing selling prices.

Independent Variable	Quantile Regression Model				
	Q 0.1	Q 0.3	Q 0.5	Q 0.7	Q 0.9
Constant	9.2542 *** (0.2197)	9.7388 *** (0.1697)	9.9254 *** (0.2004)	10.3940 *** (0.2550)	10.7078 *** (0.3947)
Age of building	−0.0301 ** (0.0149)	−0.0140 (0.0099)	−0.0122 (0.0127)	−0.0121 (0.0163)	−0.0135 (0.0217)
Total number of floors	−0.0093 (0.0196)	−0.0046 (0.0158)	−0.0085 (0.0149)	−0.0237 (0.0207)	−0.0230 (0.0288)
Parking coefficient	0.0281 (0.0250)	0.0316 * (0.0168)	0.0305 (0.0187)	0.0751 *** (0.0204)	0.1057 *** (0.0274)
Volume rate	−0.0585 *** (0.0180)	−0.0420 ** (0.0192)	−0.0297 (0.0197)	−0.0700 *** (0.0235)	−0.1156 *** (0.0391)
Green rate	0.1101 *** (0.0311)	0.0922 *** (0.0195)	0.0820 *** (0.0203)	0.0735 *** (0.0231)	0.1192 *** (0.0342)
Surrounding environment	−0.0263 *** (0.0081)	−0.0356 *** (0.0074)	−0.0371 *** (0.0091)	−0.0391 *** (0.0094)	−0.0692 *** (0.0169)
Property management	0.2363 *** (0.0528)	0.2467 *** (0.0271)	0.2298 *** (0.0249)	0.2540 *** (0.0272)	0.2941 *** (0.0327)
Cultural and sports facilities	0.0422 *** (0.0162)	0.0433 *** (0.0117)	0.0411 *** (0.0116)	0.0455 *** (0.0114)	0.0571 *** (0.0171)
Living facilities	0.0801 ** (0.0321)	0.0396 (0.0256)	0.0465 (0.0306)	−0.01242 (0.0327)	−0.0406 (0.0364)
Educational facilities	0.0576 *** (0.0174)	0.0583 *** (0.0148)	0.0558 *** (0.0142)	0.0537 *** (0.0160)	0.0697 ** (0.0350)
Bus	−0.0061 (0.0081)	−0.0208 *** (0.0072)	−0.0284 *** (0.0067)	−0.0366 *** (0.0094)	−0.0540 *** (0.0130)
Subway	−0.0595 *** (0.0110)	−0.0651 *** (0.0054)	−0.0649 *** (0.0067)	−0.0662 *** (0.0071)	−0.0725 *** (0.0122)
Shopping mall	−0.0023 (0.0091)	−0.0115 * (0.0068)	−0.0122 * (0.0073)	−0.0216 *** (0.0083)	−0.0228 ** (0.0089)
Distance to Tianfu Square	−0.2598 *** (0.0403)	−0.3003 *** (0.0442)	−0.2566 *** (0.0526)	−0.2384 *** (0.0753)	−0.1297 (0.1044)
Distance to Chunxi Road	−0.0159 (0.0425)	−0.0200 (0.0449)	−0.0300 (0.0509)	−0.0492 (0.0720)	−0.1441 (0.0920)
Air quality index	−0.2765 *** (0.0929)	−0.4242 *** (0.0786)	−0.4477 *** (0.0913)	−0.5572 *** (0.1180)	−0.7077 *** (0.2082)
Pseudo R-squared	0.5388	0.5987	0.5905	0.5416	0.4735
Quasi-LR statistic	1254.2874	2774.9934	3054.3919	2171.9346	1048.5017

Note: * *p* < 0.1, ** *p* < 0.05, *** *p* < 0.01, the value in parentheses is the standard error of the variable.
